# Complete Genome Sequences of Cluster P1 and Cluster C1 Mycobacterium smegmatis Phages Jung and Ronan

**DOI:** 10.1128/MRA.00678-20

**Published:** 2020-08-20

**Authors:** Richard Van, William Nie, Feruz Abdela, Bardia Eivazi, Dolores Kickbusch, Michael Finkle, Cody Cris, Matthew Rubinstein, Baylor Akavan, Mahdeed Raja, Jessica Vergara, Wilson Andrade, Abimael Barajas, Jocelyn Sanchez, Maria Duenas, Jay Barilla, Kurt Regner, Christy Strong, Philippos K. Tsourkas

**Affiliations:** a School of Life Sciences, University of Nevada, Las Vegas, Nevada, USA; DOE Joint Genome Institute

## Abstract

We present the complete genome sequences of Mycobacterium smegmatis phages Jung and Ronan, isolated from soil in Las Vegas, Nevada. The phages were isolated and annotated by students enrolled in a course for undergraduate research experience (CURE). Jung is a cluster P1 mycobacteriophage, while Ronan is in cluster C1.

## ANNOUNCEMENT

The soil-dwelling, acid-fast bacterium Mycobacterium smegmatis is a popular tool for courses for undergraduate research experience (CURE), such as the Howard Hughes Medical Institute’s (HHMI) Science Education Alliance Phage Hunters Advancing Genomics and Evolutionary Science (SEA-PHAGES) program, on account of its nonpathogenicity, versatility, and ease of cultivation (1). As a result, phages that infect M. smegmatis account for the largest number of sequenced phage genomes, numbering approximately 1,900 (2). In a recent high-profile case, three M. smegmatis phages isolated by students in the SEA-PHAGES program were used to treat a potentially lethal infection of antibiotic-resistant Mycobacterium abscessus in a cystic fibrosis patient (3). Here, we present the complete genomes of two M. smegmatis phages isolated by students enrolled in the SEA-PHAGES-affiliated Phage Discovery course (BIOL 207 and BIOL 217) at the University of Nevada, Las Vegas (UNLV). This was the third time the Phage Discovery course was offered at UNLV; five M. smegmatis phages have been annotated and published from the two previous offerings of the course (4, 5), as have an additional four phages that infect Paenibacillus larvae that were isolated outside the course (6).

The phages were isolated in September 2019 from garden soil from UNLV Community Gardens by students enrolled in the BIOL 207 course. Environmental samples were incubated with enrichment broth and shaken (250 rpm, 2 h) at room temperature, followed by centrifugation and filter sterilization (0.22-μm filter) of the supernatant as specified in the HHMI SEA-PHAGES Phage Discovery Guide (https://seaphages.org/faculty/information/#phagediscovery). The phages were purified and amplified in M. smegmatismc^2^155. M. smegmatis mc^2^155 was grown in Middlebrook 7H9 liquid and agar plates at 37°C as described in the Phage Discovery Guide. Liquid cultures were incubated in a tabletop shaker (37°C). Phages were purified by picking plaques with sterile pipettor tips added to 100 μl of phage buffer followed by 10-fold serial dilutions; 10 μl of each dilution was added to 500 μl of bacterial culture and sat undisturbed for 10 min at room temperature. After the addition of 4.0 ml of top agar, the solution was poured evenly over the 7H9 agar plate. Plaque size, morphology, and titer (PFU/ml) were noted after each purification round. The phage was considered purified after three rounds, producing a consistent plaque size and morphology with no sign of bacterial contamination.

Phage DNA was extracted as described in the manufacturer’s protocol in the phage DNA isolation kit (catalog number 46800; Norgen Biotek). Phage DNA was sequenced at the University of Pittsburgh. Sequencing libraries were prepared from genomic DNA using the NEB Ultra II kit. Libraries were sequenced with an Illumina MiSeq system, producing 150-bp single-end reads sufficient to provide 1,084× coverage for Jung and 273× coverage for Ronan. The reads were quality trimmed and assembled *de novo* using Newbler version 2.9 with default settings, generating a single contig which was checked for completeness, accuracy, and phage genomic termini using Consed version 29 as described in reference 7.

The phages’ GenBank and SRA accession numbers and the assembly results (coverage depth, genome length, GC content, number of genes) are listed in Table 1. Phages were assigned to a cluster based on genomic sequence similarity using the PhagesDB.org database and the Phamerator software with default settings (2, 8). Despite their identical geographic locations, the phages are not closely related; Jung is in cluster P1, while Ronan is in cluster C1. Jung is predicted to use the “cohesive ends with 3′ overhangs” DNA packaging strategy (9), while Ronan is predicted to use circularly permuted genome ends.

**TABLE 1:** Phage GenBank and SRA accession numbers and genome assembly results

Phage name	GenBank accession no.	SRA accession no.	Avg coverage (×)	Cluster	Genome length (bp)	GC content (%)	No. of genes
Jung	MT498061	SRX8474472	1,084	P1	46,561	67.1	77
Ronan	MT553335	SRX8474473	273	C1	154,852	64.6	264

The assembled genomes were annotated with DNA Master version 5.23.2., as described in references 10 and 11, by students enrolled in the BIOL 217 course in spring 2020. We identified 77 genes in Jung and 264 in Ronan, 34 of which are tRNAs. Protein functions were assigned using protein BLAST (https://blast.ncbi.nlm.nih.gov/Blast.cgi?PAGE=Proteins), batch CD-Search (https://ncbi.nlm.nih.gov/Structure/bwrpsb/bwrpsb.cgi), and HHPred (https://toolkit.tuebingen.mpg.de/tools/hhpred) with default settings. Using a cutoff E value of 1e-7, we assigned putative functions to 37 genes in Jung (48%) and 67 non-tRNA genes in Ronan (29%). A portal protein, a major capsid protein, two tail assembly chaperones, a tail tape measure protein, holin, lysin A and lysin B, and immunity repressor were identified in both phages. A small and large terminase, major tail protein, excise, and integrase were identified in Jung but not in Ronan. In Ronan, we identified a single 3,024-bp-long terminase but no separate large or small terminase subunits. Ronan also contains a gene that spans the genome ends and 34 tRNA genes, both of which are common features of cluster C1 mycobacteriophages.

### Data availability.

The GenBank and SRA accession numbers are listed in Table 1.
