# Correction for Van et al., “Complete Genome Sequences of Cluster P1 and Cluster C1 Mycobacterium smegmatis Phages Jung and Ronan”

**DOI:** 10.1128/MRA.00233-21

**Published:** 2021-04-29

**Authors:** Richard Van, William Nie, Feruz Abdela, Bardia Eivazi, Dolores Kickbusch, Michael Finkle, Cody Cris, Matthew Rubinstein, Baylor Akavan, Mahdeed Raja, Jessica Vergara, Wilson Andrade, Abimael Barajas, Jocelyn Sanchez, Maria Duenas, Jay Barilla, Kurt Regner, Christy Strong, Philippos K. Tsourkas

**Affiliations:** a School of Life Sciences, University of Nevada, Las Vegas, Nevada, USA

## AUTHOR CORRECTION

Volume 9, no. 34, e00678-20, 2020, https://doi.org/10.1128/MRA.00678-20. The article byline should read as given in this correction.

